# Off-pump versus on-pump coronary artery bypass grafting in patients with chronic obstructive pulmonary disease: a systematic review and meta-analysis

**DOI:** 10.1007/s11748-025-02116-3

**Published:** 2025-01-23

**Authors:** Anelise Poluboiarinov Cappellaro, Luiz F. Costa de Almeida, Manoela Lenzi Pinto, Marcelo Albuquerque Barbosa Martins, Augusto Graziani e Sousa, Júlia Gonçalves Gadelha, Ana Carolina Putini Vieira, Luís Fernando Rosati Rocha, Myat Soe Thet

**Affiliations:** 1 Centro Universitário Maurício de Nassau de Barreiras, Barreiras, Brazil; 2 Department of Surgery, Federal Fluminense University, Niterói, Brazil; 3 Universidade Federal de Ciências da Saúde de Porto Alegre, Porto Alegre, Brazil; 4 Universidade Federal de Ouro Preto, Ouro Preto, Brazil; 5 Centro Universitário de Anápolis, Anápolis, Brazil; 6 Afya Faculdade de Ciências Médicas da Paraíba, João Pessoa, Brazil; 7 Universidade Santo Amaro, São Paulo, Brazil; 8 Department of Surgery & Cancer, Imperial College London, South Kensington, United Kingdom

**Keywords:** Coronary artery bypass grafting, Chronic obstructive pulmonary disease, Off-pump, On-pump, Meta-analysis

## Abstract

**Introduction:**

Off-pump coronary artery bypass graft surgery (OPCAB) has been suggested as superior to on-pump coronary artery bypass graft surgery (ONCAB) in certain high-risk subgroups, but its benefit in patients with chronic obstructive pulmonary disease (COPD) remains controversial. This meta-analysis aimed to evaluate OPCAB versus ONCAB outcomes in COPD patients.

**Methods:**

We followed PRISMA guidelines and searched PubMed, Embase, and the Cochrane Library in August 2024 for studies comparing OPCAB and ONCAB in COPD patients. Statistical analysis was conducted using Review Manager 5.4.1 and Rstudio with a fixed or random effects model.

**Results:**

Six studies with a total of 1,687 patients were included, of which 1,062 (62.95%) underwent OPCAB. The mean patient age was 63.6 years. OPCAB did not significantly affect all-cause mortality compared to ONCAB (OR 1.14; 95% CI 0.65–1.99). There were no significant differences in reintubation (OR 0.81; 95% CI 0.53–1.23), prolonged ventilation (OR 0.54; 95% CI 0.24–1.22), post-operative atrial fibrillation (OR 0.90; 95% CI 0.70–1.15), or ARDS (OR 0.43; 95% CI 0.14–1.33). However, ventilation time was significantly shorter in the OPCAB group (MD – 5.30 h; 95% CI – 7.22 to – 3.38).

**Conclusion:**

OPCAB is associated with reduced ventilation time in COPD patients though it shows no significant difference in all-cause mortality or other post-operative complications compared to ONCAB.

**Supplementary Information:**

The online version contains supplementary material available at 10.1007/s11748-025-02116-3.

## Introduction

Chronic obstructive pulmonary disease (COPD) is recognized as the third leading cause of death worldwide [1, 2]. COPD impairs airflow and leads to prolonged exposure to harmful particles and gasses, resulting in significant respiratory symptoms [3–6]. Patients with COPD face a higher risk of post-operative complications, such as respiratory failure, pneumonia, atrial fibrillation (AF), and acute respiratory distress syndrome (ARDS) [7–10]. COPD also significantly increases morbidity and mortality in major surgical procedures like coronary artery bypass grafting (CABG) [11–13].

Traditionally, CABG is performed using cardiopulmonary bypass (CPB), while ensuring stable surgical conditions, can lead to systemic inflammation and complications, particularly in COPD patients [14–16]. Off-pump coronary artery bypass grafting (OPCAB) has been proposed as an alternative, potentially reducing inflammation, clotting issues, and the need for blood transfusions by avoiding CPB [17].

However, current studies comparing OPCAB and on-pump CABG (ONCAB) in COPD patients are limited and controversial [18–21]. While some evidence suggests that OPCAB may offer better outcomes for these patients, other studies report no significant differences, and the debate regarding its relative benefits continues [19, 22, 23]. Building on the premise that OPCAB could reduce respiratory complications by avoiding cardiopulmonary bypass, and given the limited and conflicting evidence alongside the critical need to optimize treatment for patients with severe comorbidities, we conducted a systematic review and meta-analysis to evaluate whether OPCAB is superior to ONCAB in terms of mortality and respiratory outcomes in patients with COPD.

## Methods

This systematic review and meta-analysis was performed and reported in accordance with the Cochrane Collaboration Handbook for Systematic Review of Interventions and the Preferred Reporting Items for Systematic Reviews and Meta-Analysis (PRISMA) Statement guidelines [24, 25]. A protocol was previously registered in an online database (CRD42024588116).

### Search strategy and data extraction

We conducted a systematic search of PubMed, Embase, and Cochrane Central using the following terms: ('chronic obstructive pulmonary disease' OR 'COPD' OR 'chronic bronchitis' OR 'emphysema' OR 'chronic airflow limitation') AND ('Off-Pump' OR 'On-Pump' OR 'Off-Pump Coronary Artery Bypass Grafting' OR ‘OPCAB’ OR ‘ONCAB’ OR 'On-Pump Coronary Artery Bypass Grafting' OR 'off-pump coronary surgery' OR 'on-pump coronary surgery' OR 'off-pump CABG' OR 'on-pump CABG' OR 'beating-heart surgery' OR 'cardiopulmonary bypass' OR 'CPB' OR 'minimally invasive direct coronary artery bypass' OR 'MIDCAB'). We also manually searched the references of included studies, previous systematic reviews, and gray literature for additional relevant studies. Two authors (A.C. and L.A.) independently screened all records, while two other authors (M.M. and M.P.) independently extracted data according to predefined search criteria and quality assessment protocols. Any discrepancies were resolved through discussion during a consensus meeting among the authors.

### Eligibility criteria

The inclusion criteria were restricted to studies that fulfilled all of the following requirements: (1) comparisons between OPCAB and ONCAB; (2) enrollment of patients with COPD; and (3) reporting at least one of the following outcomes: all-cause mortality, post-operative atrial fibrillation, renal complications, ARDS, number of patients undergoing prolonged ventilation (> 48 h), duration of ventilation, reintubation, reexploration for bleeding, number of grafts, and atelectasis. Studies were excluded if they had (1) overlapping patient populations; (2) no control group; or (3) were of inappropriate publication types. The primary outcome assessed was all-cause mortality.

### Quality assessment

We used the Cochrane Collaboration tool (RoB 2) to assess the risk of bias in randomized clinical trials and evaluate the quality of the studies [26]. Each trial was individually rated as having a Low, Some Concerns, or High risk of bias across five domains: selection, performance, detection, attrition, and reporting biases. This process was conducted independently by two authors (A.P.C and J.G.G), and any conflicts were resolved by consensus.

Additionally, the Newcastle–Ottawa Scale (NOS) was used to assess the quality of non-randomized studies [27]. The studies were evaluated based on the representativeness of the exposed cohort, the selection of the non-exposed cohort, and the measurement of outcomes. We classified studies with a score of 7 to 9 as low risk, those with a score of 4 to 6 as moderate risk, and those with a score below 4 as high risk. This process was also carried out by two independent authors, with any disagreements resolved by consensus.

### Statistical analysis

The odds ratio (OR) was used to aggregate binary outcomes into a 95% confidence interval using the Restricted Maximum Likelihood (REML) method. Continuous outcomes were synthesized using a pooled mean difference (MD). We used fixed effects models for outcomes with heterogeneity less than 50% and random effects models for outcomes with heterogeneity greater than 50%. Heterogeneity was assessed with the Cochran Q test and I^2^ statistics, where an I-square > 50% and a p-value less than 0.05 were considered significant [28]. Statistical analyses were performed using Review Manager 5.4.1 (Cochrane Centre, The Cochrane Collaboration, Denmark) and RStudio 2023.12.1. Publication bias was evaluated through funnel plot analysis of endpoints, and Egger’s test was used to assess funnel plot asymmetry by performing a linear regression of the log risk ratio against the inverse of the standard error.

## Results

### Baseline characteristics of included studies

**Fig. 1:**
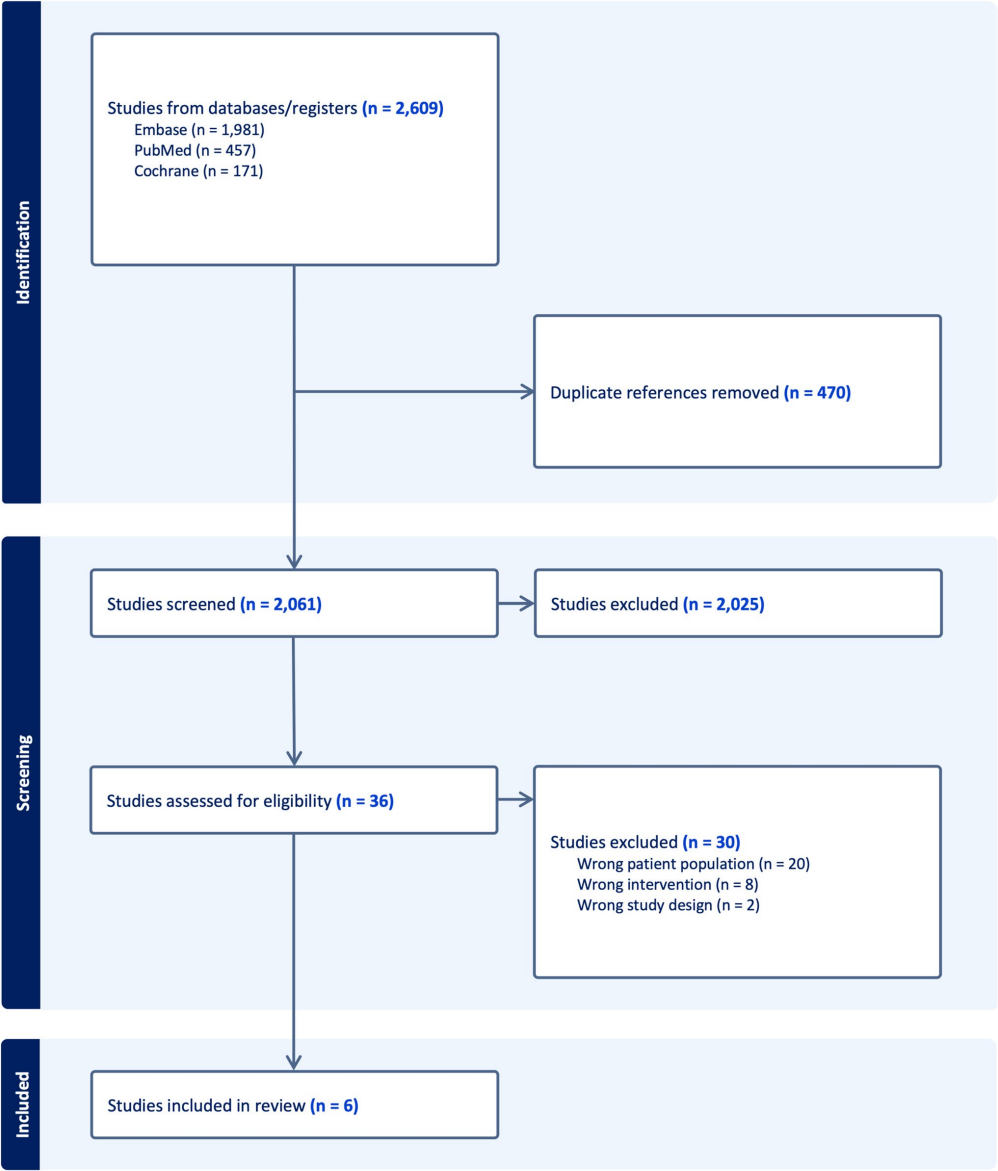
Preferred Reporting Items for Systematic Reviews and Meta-Analyses (PRISMA) flow diagram for literature search and selection

As illustrated in Fig. 1, the search generated 2531 results across PubMed, Embase, and Cochrane. After removing duplicates and irrelevant articles, 36 studies remained for full review to determine their eligibility based on the inclusion criteria. Of these, 6 studies were selected, encompassing a total of 1687 patients [19, 22, 23, 29–31]. We found three studies through searches in the gray literature and non-traditional databases using the database search strategy. The included patients had a mean age of 63.6 years, with 63% (1062 out of 1687) receiving OPCAB as the intervention. Study characteristics are detailed in Table 1.

**Table 1:** Baseline characteristics of included studies

Author, year	Study Design	Country	Number of patients	Age (mean)	Men (%)	Hypertension (%)	Diabetes (%)	Smokers (%)
Almassi 2013	RCT	USA	220/238	63.5/64	N/A	192/210	95/103	114/102
Ammar 2011	Cohort	Egypt	31/31	68/68	23/23	0/31	14/12	31/4
Kerendi 2011	Cohort	USA	720/264	65.6/63.4	446/169	609/224	276/108	294/108
Mady 2023	RCT	Egypt	30/30	56.5	N/A	N/A	N/A	N/A
Ya-bing 2004	RCT	China	28/19	62.4/61.3	12/7	7	6/4	20/15
Yokoyama 2000	Cohort	USA	33/43	67/68	N/A	N/A	N/A	N/A

*N/A* Not available; *RCT* Randomized controlled trial

### Quality assessment

The Risk of Bias 2 (RoB 2) was used for quality assessment in randomized studies. Ya-bing (2004) was the only study labeled as having a high risk of bias due to concerns in the domains of ‘randomization process’, ‘deviations from intended interventions’, ‘measurement of the outcomes’ and ‘selection of the reported results’. The other studies, Almassi 2013 and Mady 2023 were considered at ‘some concerns’ risk of bias (Table S6). To analyze the quality of non-randomized studies, the Newcastle–Ottawa Scale (NOS) was used. All observational articles were classified as low risk of bias, with scores ranging from 8 to 9 (Table S7).

### Outcomes of OPCAB vs ONCAB in patients with COPD

**Fig. 2:**
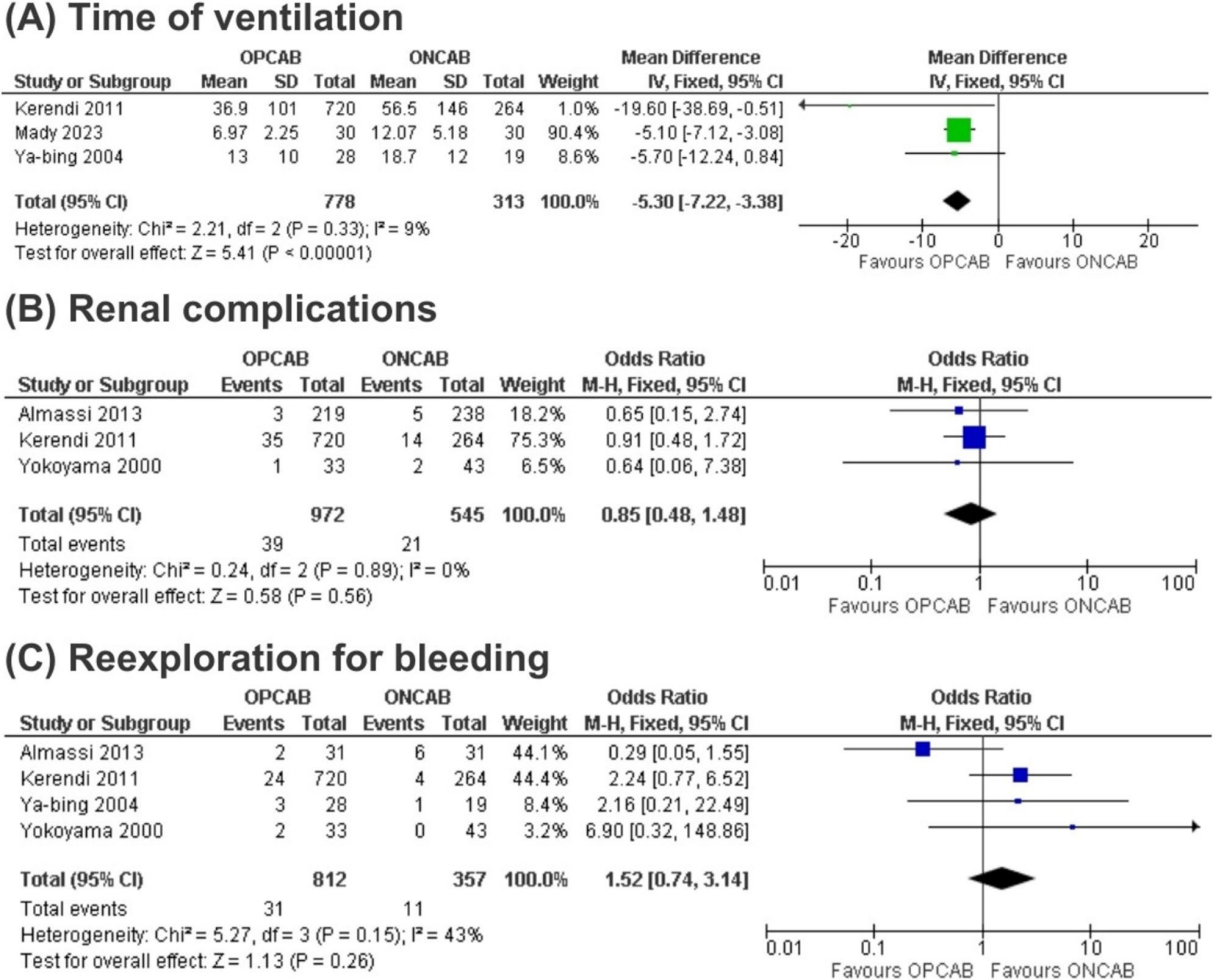
Forest plots for comparing off-pump coronary artery bypass (OPCAB) versus on-pump coronary artery bypass (ONCAB) across three outcomes: **A** Time of ventilation, **B** Renal complications and **C** Reexploration for bleeding in patients with Chronic Obstructive Pulmonary Disease (COPD)

Ventilation time in hours was significantly shorter in the OPCAB group compared to the ONCAB approach in patients with COPD (MD – 5.30; 95% CI – 7.22, – 3.38; *p* < 0.05; i2 = 9%) (Fig. 2). Additionally, the number of grafts in surgery was also significantly lower in the OPCAB group (MD – 0.24; 95% CI – 0.47, – 0.01; *p* < 0.05; i2 = 0%). There were no significant differences between the compared groups in atelectasis (OR 0.79; 95% CI 0.20–3.07; *p* = 0.73; I2 = 11%), number of patients undergoing prolonged ventilation (> 48 h) (OR 0.54; 95% CI 0.24–1.22; *p* = 0.14; I2 = 65%), and reintubation (OR 0.81; 95% CI 0.53–1.23; *p* = 0.31; I2 = 0%).

**Fig. 3:**
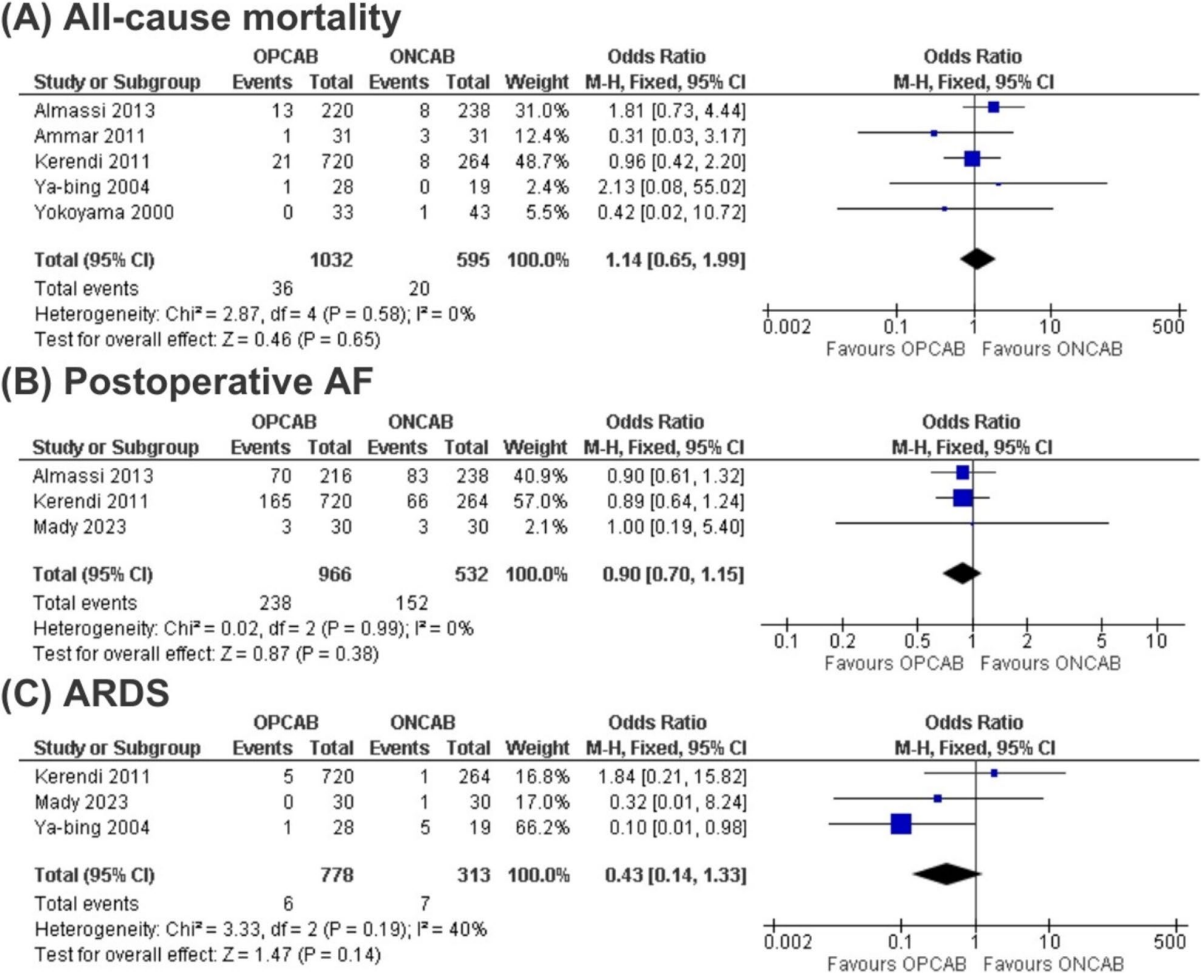
Forest plots for comparing off-pump coronary artery bypass (OPCAB) versus on-pump coronary artery bypass (ONCAB) across three outcomes: **A** All-cause mortality, **B** Postoperative atrial fibrillation (AF) and **C** Acute Respiratory Distress Syndrome (ARDS) in patients with Chronic Obstructive Pulmonary Disease (COPD)

The OPCAB approach did not show significant differences in all-cause mortality when compared to ONCAB in patients with chronic lung disease (OR 1.14; 95% CI 0.65–1.99; *p* = 0.62; I2 = 0%) (Fig. 3). We conducted a sensitivity analysis using random-effects models due to low heterogeneity, and the results were consistent with the findings (OR 1.16; 95% CI 0.65–2.04). Additionally, a subgroup analysis including only randomized studies was conducted in comparison with observational studies, and the results remained consistent (supplementary data). Furthermore, there were no differences between the compared approaches for post-operative atrial fibrillation (OR 0.90; 95% CI 0.70–1.15; *p* = 0.38; I2 = 0%), ARDS (OR 0.43; 95% CI 0.14–1.33; *p* = 0.14; I2 = 40%), Reexploration for bleeding (OR 1.52; 95% CI 0.74–3.14; *p* = 0.26; I2 = 43%) and renal complications (OR 0.85; 95% CI 0.48–1.48; *p* = 0.56; I2 = 0%). A sensitivity analysis comparing random effects and fixed-effects models for outcomes with low heterogeneity is presented in Table S8. The results demonstrated consistency when assessed using the random effects model.

### Publication bias assessment

Funnel plot analysis for the outcome of all-cause mortality did not show any evidence of publication bias. The studies occupied a relatively symmetric distribution according to individual weight and converged toward the pooled effect as the weight increased. The exclusion of Mady 2023 from the funnel plot was due to the unavailability of the necessary data for its inclusion in the all-cause mortality analysis.

## Discussion

This systematic review and meta-analysis, involving 1687 participants, evaluated the impact of off-pump versus on-pump coronary artery bypass grafting in patients with chronic obstructive pulmonary disease. Our main findings are as follows: (1) OPCAB was associated with a significantly shorter ventilation time compared to ONCAB; (2) OPCAB required fewer grafts; and (3) no significant differences were observed in all-cause mortality between the two approaches.

Coronary artery bypass grafting (CABG) is a well-established therapy renowned for its effectiveness in enhancing patients' quality of life and prolonging survival [32, 33]. The traditional CABG approach, which utilizes cardiopulmonary bypass (CPB), is frequently preferred for its capacity to achieve comprehensive revascularization [34–36]. However, the comparative efficacy of CABG with CPB versus off-pump CABG (OPCAB) in patients with chronic obstructive pulmonary disease (COPD) remains contested [37, 38]. Given the high prevalence of COPD and its detrimental impact on post-operative complications, it is crucial to adopt strategies that minimize both respiratory and systemic issues [39]. Thus, meticulous selection of the surgical approach and effective management of risk factors are essential for optimizing clinical outcomes in this high-risk population.

Postoperative pulmonary complications in COPD patients are associated with prolonged hospital stays, increased use of resources, and higher hospital costs [40, 41]. However, our data suggest that OPCAB may improve post-operative pulmonary outcomes by reducing the average ventilation time by 5 h. This reduction may help mitigate the trauma and infections often associated with the time of ventilation [41]. OPCAB likely offers this benefit by avoiding the systemic inflammatory response triggered by cardiopulmonary bypass, which generally results in increased pulmonary vascular permeability and impaired gas exchange [42]. Another hypothesis for the reduced ventilation time in the OPCAB group is that the number of grafts performed during surgery may directly affect the duration of mechanical ventilation. More extensive or complex procedures, which involve a greater number of grafts, are typically associated with longer operation times and may require an extended period of ventilation for optimal recovery [46, 48]. Additionally, no significant differences were found in all-cause mortality or other post-operative cardiovascular outcomes between the groups, underscoring the viability of OPCAB as an approach that offers advantageous post-operative pulmonary results. Therefore, OPCAB emerges as a viable alternative for reducing post-operative pulmonary morbidity in patients with COPD.

Furthermore, our results indicated a reduced average number of grafts in patients undergoing OPCAB, which is consistent with previously published studies [43–45]. However, it is now understood that this difference may be primarily attributed to the selection of patients who require fewer grafts for OPCAB, rather than a more efficient use of grafts [46]. This situation is likely attributable to the technical challenges involved in performing the procedure [46]. Therefore, it is recommended that OPCAB be performed by more experienced surgeons, as incomplete revascularization is linked to worse outcomes for patients [46, 47].

Our study has limitations. First, only three of the included studies are randomized, which may introduce confounding factors. Additionally, the relatively small number of patients in each study may limit the generalizability of our results. Furthermore, there was considerable heterogeneity observed in some outcomes, which could impact the interpretation and robustness of the conclusions. However, we employed a random effects analysis as a conservative measure to address the heterogeneity among the studies. We also conducted a leave-one-out sensitivity analysis to reinforce the robustness of our findings. Lastly, we performed a subgroup analysis distinguishing between randomized and observational studies, which provided confirmatory results and supported our conclusions.

## Conclusion

This meta-analysis assessed 1687 COPD patients undergoing off-pump (OPCAB) versus on-pump (ONCAB) coronary artery bypass grafting. The results revealed that the OPCAB group had significantly shorter ventilation times. However, no significant differences were found between the two groups regarding all-cause mortality or other clinical outcomes. Therefore, our findings support the conclusion that there are no clinically relevant differences between the approaches in patients with COPD.

## Supplementary Information

Below is the link to the electronic supplementary material.

Supplementary file1 (DOCX 76 KB)

## Data Availability

The datasets generated and/or analyzed during the current study are available from the corresponding author upon request.

## Abbreviations

ARDS: Acute Respiratory Distress Syndrome
AF: Atrial Fibrillation
CPB: Cardiopulmonary bypass
COPD: Chronic Obstructive Pulmonary Disease
CABG: Coronary artery bypass grafting
MD: Mean difference
NOS: Newcastle–Ottawa Scale
OR: Odds ratio
OPCAB: Off-pump coronary artery bypass graft surgery
ONCAB: On-pump coronary artery bypass graft surgery
PRISMA: Preferred Reporting Items for Systematic Reviews and Meta-Analysis
RCT: Randomized controlled trial
REML: Restricted Maximum Likelihood
RoB 2: Risk of Bias 2
