# Supplementary material for: Off-pump versus on-pump coronary artery bypass grafting in patients with chronic obstructive pulmonary disease: a systematic review and meta-analysis
Source: Gen Thorac Cardiovasc Surg. 2025 Jan 23;73(4):201–8. doi: 10.1007/s11748-025-02116-3 (PMC11914329; doi:10.1007/s11748-025-02116-3)
Supplement: Supplementary file 1 — Supplementary file1 (DOCX 76 KB) [file 11748_2025_2116_MOESM1_ESM.docx]

**Supplementary Appendix**

**Figure S1** - Fixed effects models in all-cause mortality

**Figure S2** - Leave one out for all-cause mortality

**Figure S3** - Forest plot comparing RCTs vs observational studies for all-cause mortality

**Figure S4 -** Funnel plot of all-cause mortality

**Figure S5** - Egger’s test

**Table S6 -** Risk of bias assessment in randomized trials (RoB 2)

**Figure S7** - Newcastle-Ottawa Scale (NOS) for cohort studies

**Table S8 -** Sensitivity Analysis Comparing Results Between Random-Effects and Fixed-Effects Models

**Figure S1 - Fixed effects models in all-cause mortality**

**
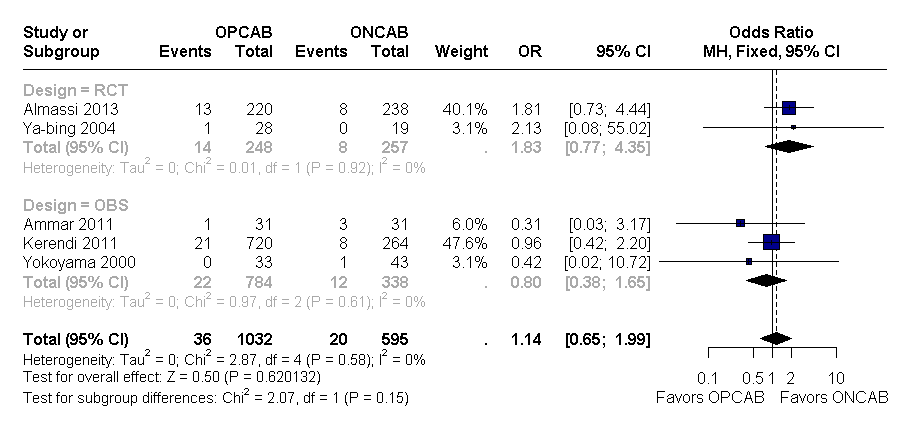
**

**Figure S2 - Leave one out for all-cause mortality**


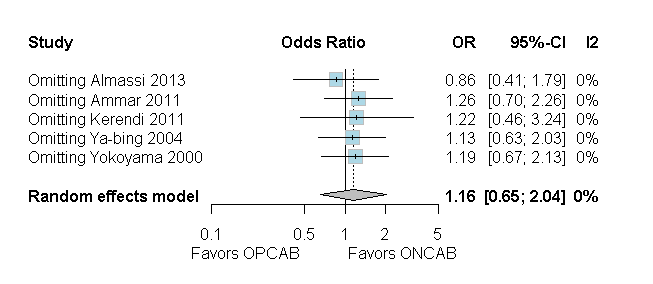


**Figure S3 - Forest plot comparing RCTs vs observational studies for all-cause mortality**


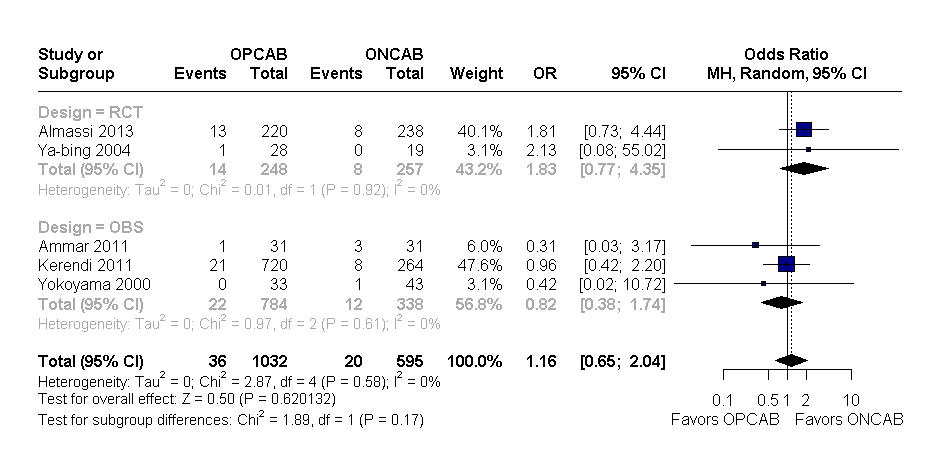


**Figure S4 - Funnel plot of all-cause mortality**

**
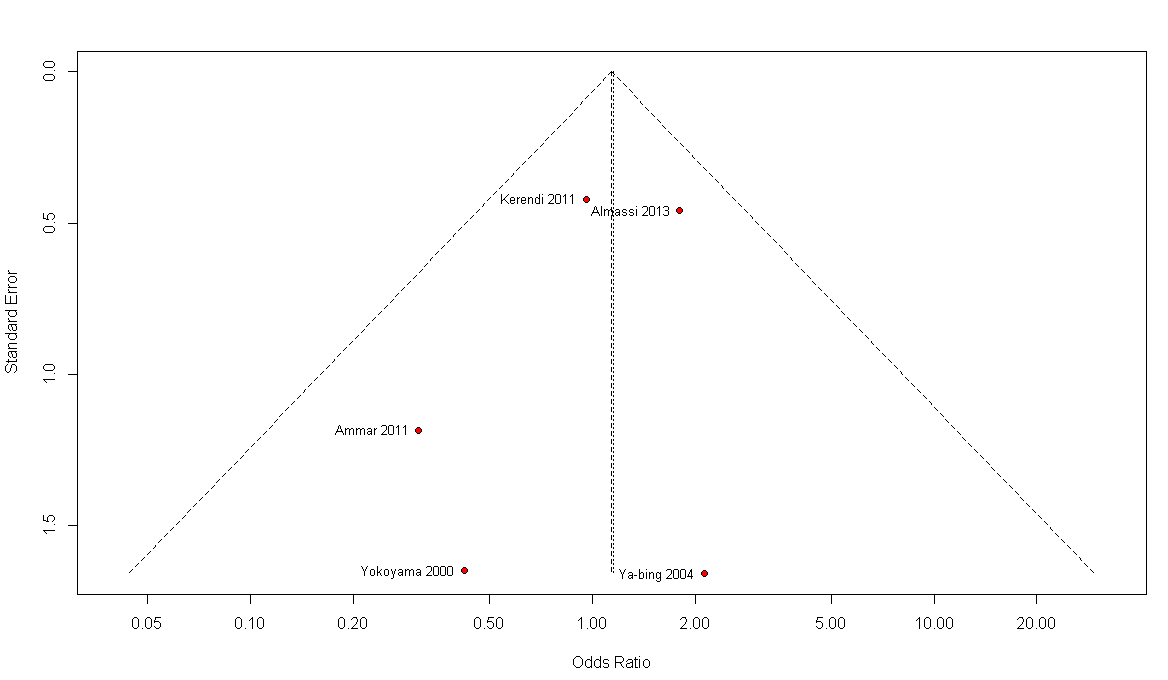
**

**Figure S5 - Egger’s test**

**
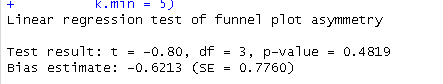
**

**Figure S6 - Risk of bias assessment in randomized trials (RoB 2)**

| **Study** | **Bias from randomization process** | **Bias due to deviations from intended interventions** | **Bias due to missing outcome data** | **Bias in measurement of the outcomes** | **Bias in selection of the reported result** | **Overall risk of bias** |
| --- | --- | --- | --- | --- | --- | --- |
| **Almassi 2013** | Some concerns | Low | Low | Low | Low | Some concerns |
| **Mady 2023** | Low | Low | Low | Low | Some concerns | Some concerns |
| **Ya-bing 2004** | Some concerns | Some concerns | Low | Some concerns | Some concerns | **High** |

**Figure S7 - Newcastle-Ottawa Scale for cohort studies**

| Authors, Year | Selection | | | | Comparability | Outcome | | | Final score |
| --- | --- | --- | --- | --- | --- | --- | --- | --- | --- |
|  | 1 | 2 | 3 | 4 | 1a/1b | 1 | 2 | 3 |  |
| Ammar 2011 | ★ | ★ | ★ | ★ | ★★ | ★ |  | ★ | 8/9 |
| Kerendi 2011 | ★ | ★ | ★ | ★ | ★★ | ★ | ★ | ★ | 9/9 |
| Yokoyama 2000 | ★ | ★ | ★ | ★ | ★ | ★ | ★ | ★ | 8/9 |

**Table S8** – **Sensitivity Analysis Comparing Results Between Random-Effects and Fixed-Effects Models**

| **Outcomes** | **Random Effects** | |  | **Fixed Effects** | |
| --- | --- | --- | --- | --- | --- |
|  | **OR (95% CI)** | **I^2^** |  | **OR (95% CI)** | **I^2^** |
| **All-cause mortality** | **1.16 (0.65; 2.04)** | **0%** |  | **1.14 (0.65; 1.99)** | **0%** |
| **ARDS** | **0.42 (0.07; 2.65)** | **40%** |  | **0.43 (0.14-1.33)** | **40%** |
| **Atelectasis** | **0.81 (0.17; 3.83)** | **11%** |  | **0.81 (0.17; 3.83)** | **11%** |
| **AF** | **0.90 (0.70; 1.15)** | **0%** |  | **0.90 (0.70; 1.15)** | **0%** |
| **Renal complication** | **0.85 (0.48; 1.50)** | **0%** |  | **0.85 (0.48; 1.48)** | **0%** |
| **Reexploration for bleeding** | **1.45 (0.42; 4.92)** | **43%** |  | **1.52 (0.74; 3.14)** | **43%** |
| **Reintubation** | **0.81 (0.53; 1.23)** | **0%** |  | **0.81 (0.53; 1.23)** | **0%** |
| **Outcomes** | **MD (95% CI)** | **I^2^** |  | **MD (95% CI)** | **I^2^** |
| **Number of grafts** | **-0.24 (-0.47; -0.01)** | **0%** |  | **-0.24 (-0.47; -0.01)** | **0%** |
| **Time of ventilation** | **-5.46 (-8.11; -2.82)** | **9%** |  | **-5.30 (-7.22; -3.38)** | **9%** |

Sensitivity analysis comparing fixed-effects and random-effects models demonstrated consistent results across all outcomes. AF - atrial fibrillation; ARDS - Acute Respiratory Distress Syndrome; MD - Mean Difference; CI - Confidence interval; OR - Odds Ratio;
